# Editorial: T-Cell Signaling Networks in Health and Disease

**DOI:** 10.3389/fimmu.2022.875580

**Published:** 2022-04-06

**Authors:** Christopher E. Rudd, Isabel Merida, William Hawse

**Affiliations:** ^1^ Department of Medicine, Université de Montréal, Montréal, QC, Canada; ^2^ Division of Immunology-Oncology, Research Center Maisonneuve-Rosemont Hospital, Montréal, QC, Canada; ^3^ Department of Immunology and Oncology, National Center for Biotechnology (CNB), Madrid, Spain; ^4^ Department of Immunology, University of Pittsburgh, Pittsburgh, PA, United States

**Keywords:** T cell, TCR (T cell receptor), signal transduction, chimeric antigen receptor modified T (CAR-T) cell, immunotherapy, adaptive immunity

Intracellular signaling networks control every aspect of T cell biology. Proper signaling promotes T cell development, cellular differentiation, adaptive immune responses and maintenance of immune homeostasis. T cell signaling and activation mechanisms play a fundamental role in disease processes. Ligation of the T-cell receptor (TCR) alone by major histocompatibility complex (MHC)-peptide or cancer neo-antigens is needed to activate T-cells. Stimulation is induced by a tyrosine phosphorylation cascade initiated by the *src* kinase p56^lck^ and its binding to the co-receptors CD4 and CD8 (1–3). p56^lck^ phosphorylates immune tyrosine activation motifs (ITAMs) on the TCR associated CD3 and zeta chains that recruit and activate a second kinase, zeta-chain-associated protein kinase 70 (ZAP-70) (4). p56^lck^ and ZAP-70 in turn then phosphorylate adaptors to form complexes or molecular switches that amplify and integrate signals for specific functions. Adaptors LAT and SLP-76 facilitate phospholipase C phosphorylation and calcium mobilization, while ADAP and SKAP1 (aka SKAP55) activate integrins (5–7). Despite this, second and third sets of signals from positive and inhibitory co-receptors (IFs) determine the final outcome of activation. CD28 and ICOS potentiate activation, while inhibitory co-receptors such as CTLA-4, PD-1, LAG3, TIGIT, TIM-3 and others limit or terminate T-cell responses (8–10). They are also expressed on exhausted or dysfunctional T-cells which develop after repeated antigenic stimulation. CD28 can both amplify TCR signaling or generate independent signals (10, 11). Subsequent signals are provided by the CD40 pathways as well as an array of cytokines to bind to receptors leading to the full development of T-cell subsets and different levels of differentiation and effector function. This work has led to the development of chimeric antigen receptor modified T cells (CAR-T) to thwart diseases while further advances in our basic understanding of the signals proteins while further improve the efficacy of CAR therapy (12, 13).

New technologies have also revolutionized our ability to better visualize the organization of signaling processes, characterize signaling cascades at the systems level and study the role of metabolites and signaling lipids in regulating T cell activation. As shown in this Research Topic, there is still much to learn about signaling in T cells and that a more in-depth basic understanding of signaling mechanisms could be leveraged to control T cell activity and rationally engineer T cell-based immunotherapies.

O’Brien et al. detail work on the spontaneous differentiation of T follicular helper cells in mice carrying a mutant form of the LAT (linker for activation of T cells) adaptor on the PLCγ1 binding site (Y136). LAT is an integral membrane adaptor protein that constitutes in T cells a major substrate of the ZAP-70 protein tyrosine kinase. They show that mice with a tyrosine to phenylalanine mutation on LAT 136 (LATY136F) have reduced TCR signaling that disrupts thymocyte development. Interestingly, peripheral CD4^+^T cells are hyper-activated. LATY136F mice develop an autoimmune-like syndrome, characterized with increased Th2 cytokines, T cell infiltration into organs, B cell activation and autoantibody production. Using mice that inducibly express LATY136F, they further showed that increased Tfh cell differentiation was likely the result of defective LAT-PLCγ1 signaling. Mechanistically, B cells were required for spontaneous Tfh cell development and T cell expansion in LATY136F mice. These results identify a role for tonic LAT-PLCγ1 signaling in modulating Tfh cell differentiation and development of an autoimmune syndrome.

Sandner et al. outlined the role the tyrosine kinase Tec in the regulation of effector Th17 differentiation and plasticity in T-cell-driven intestinal inflammation. T helper (Th) 17 cells control bacterial and fungal infections and antagonize autoimmune diseases. They outline how Th17 cells heterogeneous where plasticity to other subsets is controlled by cytokine signaling. Sandner et al. found that Th17 differentiation was enhanced by the presence of low interleukin-6 (IL-6) in absence of Tec. This correlated with increased STAT3 phosphorylation and higher levesl of *Il23r* expression. IL-17A fate mapping mouse combined with adoptive cell transfer models further showed that Tec limited Th17 effector differentiation and plasticity.

Gaunt et al. found that the MS remyelinating drug, Bexarotene (an RXR Agonist), promotes induction of human Tregs and suppresses Th17 differentiation. Murine studies demonstrated that RXR agonists enhanced all-trans-retinoic acid (*at*RA) to promote T-regulatory cell (Treg) induction and reduce Th17 differentiation *in vitro.* Bexarotene also promotes humanTreg induction, but its mode of action is independent of of *at*RA and retinoic acid receptor signaling, as is the case for murine T cells. These findings highlight that RXR agonists could be therapeutically relevant for treating autoimmune diseases.

This is then followed by an article from Khan et al. outlined the role of PP2A and its inhibitors in helper T-Cell differentiation and autoimmunity. Protein phosphatase 2A (PP2A) is a heterotrimeric Ser/Thr phosphatase that regulates the phosphorylation status of multiple proteins that function in diverse cellular processes. While PP2A is established as a tumor suppressor, there is growing evidence that PP2A functions in T cells to constrain inflammatory responses. Khan et al. present a comprehensive review on PP2A in the context of T-cell differentiation and autoimmunity. Additionally, Khan et al. detail endogenous inhibitors and small-molecule activators that modulate of PP2A phosphatase activity, which could be utilized therapeutically for multiple autoimmune disorders.

Linked to protein phosphorylation is the regulation of all-important metabolism. Peng et al. explore the issue of metabolic reprogramming and reactive oxygen species in T cell immunity. In this paper, they assess the role of metabolic reprogramming needed for the energetic and biosynthetic demands of T-cells thoughout their lifespan. In this context, multiple metabolic pathways promote the generation of reactive oxygen species (ROS). Because ROS can damage proteins, lipids and nucleic acids, the imbalance between ROS generation and scavenging systems could dysregulate T cells leading to multiple pathologies. Peng et al. discuss the connection between cellular lifespan and the metabolic programming needed to support different T cell subsets and detail mechanisms by which ROS could impact T cell activity.

Hu et al. present a bioinformatic analysis to depict CD8^+^ T-cell developmental trajectories and characterize pre-exhausted T cells isolated from colorectal cancer (CRC) patients. in the scRNA-seq data set using a dynamic network biomarker (DNB). The Dynamic Network Biomarker (DNB) identified that *CCT6A* was a biomarker for a pre-exhausted T-cell population in CRC. Both *TUBA1B* and *CCT6A* expression were associated with the overall survival of COAD patients. The results presented by Hu et al. findings provide fresh insights into T-cell exhaustion and propose approaches for targeted immunotherapy in CRC.

Xiong et al. present work on the impact human placenta MSCs-derived exosomes (hPMSC-Exo) in aging-related CD4^+^ T cell senescence. Mesenchymal stem cells (MSCs)-derived exosomes are a potential therapeutic for many aging-related diseases. Using the D-gal induced mouse aging model, Xiong et al. identified that hPMSC-Exo carries miRNA-21, which attenuates CD4^+^ T cell senescence by activating the PTEN/PI3K-Nrf2 axis to engage exogenous antioxidant defenses.

Lastly, Kunkl et al. provided a paper on the binding of Staphylococcal Enterotoxin B (SEB) super antigen to B7 receptors that could trigger TCR- and CD28-mediated inflammatory signals even in the absence of MHC class II molecules. Superantigen have a potent stimulatory activity for T lymphocytes as seen in several species where they cross-link variable parts of the T-cell receptor (TCR) with MHC class II molecules on accessory or target cells. Using this mechanism, the authors describe how they can stimulate CD4+, CD8+ and gamma delta + T cells. Depsite this, it has been unclear whether they can also bind co-stimulatory receptors such as CD28. Kunkl et al. demonstrate that MHC class II binding is dispensable for the inflammatory activity of SEB. However, SEB binding to to B7 molecules triggers TCR- and CD28-mediated inflammatory signalling. They also provide interesting evidence that SEB favours the recruitment of the TCR into the immunological synapse by strengthening the interaction between CD28 and B7.

Overall, our series has underscored the central importance of intracellular signaling networks in the control of various aspects of T cell biology and how this acquired knowledge is leading to the development of new therapeutics including the development of re-purposed therapeutics in the field. A basic understanding of the signaling events is needed for the development of new therapeutic approaches. Further, dysregulation of signaling *via* genetic mutations to T cell receptor proximal signaling proteins underscores multiple clinical pathologies.

## Author Contributions

CR and WH wrote the editoral and IM gave constructive feedback. All authors contributed to the article and approved the submitted version.

## Conflict of Interest

The authors declare that the research was conducted in the absence of any commercial or financial relationships that could be construed as a potential conflict of interest.

## Publisher’s Note

All claims expressed in this article are solely those of the authors and do not necessarily represent those of their affiliated organizations, or those of the publisher, the editors and the reviewers. Any product that may be evaluated in this article, or claim that may be made by its manufacturer, is not guaranteed or endorsed by the publisher.
